# Toxic coral gobies reduce the feeding rate of a corallivorous butterflyfish on *Acropora* corals

**DOI:** 10.1007/s00338-012-0947-3

**Published:** 2012-08-25

**Authors:** M. Dirnwoeber, J. Herler

**Affiliations:** Faculty of Life Sciences, Department of Integrative Zoology, University of Vienna, Althanstrasse 14, 1090 Vienna, Austria

**Keywords:** *Gobiodon*, *Chaetodon austriacus*, Coral-fish association, Resource defence, Selective corallivory, Red Sea

## Abstract

The obligate coral-dwelling gobiid genus *Gobiodon* inhabits *Acropora* corals and has developed various physiological, morphological and ethological adaptations towards this life habit. While the advantages of this coral-fish association are well documented for *Gobiodon*, possible fitness-increasing factors for the host coral are unknown. This study examines the influence of coral-dwelling gobies on the feeding behaviour of obligate corallivorous butterflyfishes. In an aquarium experiment using video observation, the corallivorous butterflyfish *Chaetodon austriacus* fed significantly less on corals inhabited by two *Gobiodon* species compared to unoccupied coral colonies of similar size. The more agonistic species *G. histrio*, which mostly displayed directed movements towards butterflyfishes, decreased butterflyfish bite rate by 62–98 % compared to uninhabited colonies. For *Gobiodon* sp. 3, which mostly displayed undirected movements in response to visits by *C. austriacus*, bite rate reduction was 64–68 %. The scale-less skin of *Gobiodon* spp. is covered by mucus that is toxic and multi-functional by reducing predation as well as affecting parasite attachment. A choice flume experiment suggests that the highly diluted skin mucus of *Gobiodon* spp. also functions as a corallivore repellent. This study demonstrates that *Gobiodon* spp. exhibit resource defence against coral-feeding butterflyfishes and also that coral colonies without resident *Gobiodon* suffer higher predation rates. Although the genus *Gobiodon* is probably a facultative corallivore, this study shows that by reducing predation on inhabited colonies by other fishes, these obligate coral-dwellers either compensate for their own fitness-decreasing impact on host colonies or live in a mutualistic association with them.

## Introduction

Corallivory is a widespread and important feeding mode among coral reef fishes, exhibited mostly by butterflyfishes (Cole et al. 2008). Adult butterflyfishes that feed exclusively on corals each remove up to 3 g of coral tissue per day (Cole et al. 2011). Although this amount is scattered across different colonies within butterflyfish territories, the effect of coral tissue removal implies fitness consequences for coral colonies because energy must be invested in tissue regeneration rather than in growth and reproduction (see review by Rotjan and Lewis 2008). The net drain of energy through tissue removal on single colonies is further increased because corallivorous butterflyfishes typically consume only a small spectrum of available coral species, particularly of the genus *Acropora* (Alwany et al. 2003; Berumen et al. 2005; Pratchett 2005). Although *Acropora* corals are generally prevalent and fast growing, their vulnerability to bleaching has reduced their relative abundance in many locations. Accordingly, within a recovering reef, the average *Acropora* colony is younger and hence smaller than before bleaching events (McClanahan et al. 2008). Territorial butterflyfishes avoid feeding on very small and juvenile colonies (Niedermüller et al. 2009; Cole and Pratchett 2011b). This is likely to further increase the chronic predation pressure on less abundant and more fertile, medium- and large-sized *Acropora* colonies. The result is an intensified negative effect on colony fitness (Gochfeld 2010; Cole and Pratchett 2011a) contributing to the persistent stress on such colonies from several other threats, including climate change (Cole et al. 2011). Understanding and identifying the factors that shape selective feeding preferences in butterflyfishes are therefore crucial for determining the impact of butterflyfish feeding on coral communities.

Selective feeding by butterflyfishes on species of *Acropora* has been documented in the Indo-Pacific region (e.g., Berumen et al. 2005) and Red Sea (e.g., Alwany et al. 2003; Niedermüller et al. 2009). Mechanisms underlying selective feeding are diverse and operate between and within coral species as well as on single colonies (Tricas 1989; Berumen et al. 2005; Berumen and Pratchett 2006; Chong-Seng et al. 2010; Cole and Pratchett 2011b). Among these mechanisms, reduced corallivory on single colonies can also be caused by more subtle, although significant, effects of associated organisms, such as farming damselfishes that defend their territories and the corals contained in their territories against intruding butterflyfishes (Gochfeld 2010; Johnson et al. 2011). Similarly, coral-dwelling fishes of the gobiid genus *Gobiodon*, which live exclusively in *Acropora* corals, have been suggested to influence selective corallivory in butterflyfishes (Lassig 1981; Niedermüller et al. 2009). Although coral-dwelling organisms can increase coral colony fitness, the associations between these highly specialised and obligatorily coral-dwelling fishes and their host colonies have not yet been experimentally determined at the species-specific level.

Several other coral-dwelling organisms significantly contribute to the fitness of their coral hosts through various mechanisms. They maintain coral health by clearing sediment (Stewart et al. 2006), defend corals against coral predators (Weber and Woodhead 1970; Godwin and Fautin 1992; Pratchett 2001), fertilise the corals’ endosymbiotic algae through their nutrient-rich faeces (Porat and Chadwick-Furman 2005; Holbrook et al. 2008) and increase interstitial oxygen levels by circulating water through the coral colony through sleep-swimming movements (Goldshmid et al. 2004). Through such mechanisms, coral-associated species can enhance coral growth, survival, long-term reproductive output and the density of zooxanthellae, as well as reduce the amount of energy allocated to tissue regeneration.

Among the fishes with strongest affinity and reliance on scleractinian corals are coral-dwelling gobies of the genus *Gobiodon*. Although the interstitial shelter of corymbose and digitate *Acropora* corals are vital for these coral inhabitants, whether this association is beneficial for inhabited coral colonies remains largely unknown. *Gobiodon* spp. are highly specialised, exhibiting habitat choice at the level of coral species (Munday et al. 1997; Dirnwoeber and Herler 2007). The diversification of *Gobiodon* began approximately 10 million years ago (Herler et al. 2009), which coincides with the diversification of common host coral species (van Oppen et al. 2001). Various physiological, morphological and ethological adaptations towards a coral-dwelling lifestyle have been documented (Nakashima et al. 1996; Munday et al. 1998, 2006; Nilsson et al. 2004; Herler 2007). Accordingly, adult breeding pairs would be expected to avoid migrations (Wall and Herler 2008) and should benefit from maintaining healthy coral colonies. Adaptations include the possession of crinotoxic epidermal gland cells and a thick mucus epidermis instead of scales (Hashimoto et al. 1974). The compounds of this skin mucus are easily soluble in water (Hashimoto et al. 1974) and cause food refusal in predators and avoidance responses in parasites (Munday et al. 2003; Schubert et al. 2003). This general mucus function as a predator/parasite deterrent may also extend to the close proximity of the inhabited colony. Agonistic behaviour of gobies towards corallivores would be more effective if supplemented by such deterrent functions, as suggested by Lassig (1981).

Resource defence against corallivorous butterflyfishes and other potential corallivorous fishes would be highly beneficial for both the gobies and their host corals. We hypothesise that colonies benefit from being inhabited by these highly specialised reef fishes because they reduce the predation rate by corallivorous butterflyfishes and thus influence their feeding preferences. Our experimental ex situ approach excluded confounding variables such as territory size, conspecifics, coral species composition and abundance as well as colony size, all of which influence butterflyfish feeding (Cole et al. 2008; Niedermüller et al. 2009; Cole and Pratchett 2011b). This study was designed to assess the association between *Gobiodon* and their host corals by investigating and quantifying the effect of two *Gobiodon* species on the feeding behaviour of the obligate corallivorous butterflyfish *Chaetodon austriacus*. The focus was on medium-sized colonies, and the behaviour of *Gobiodon* was incorporated. The two goby species, the conspicuously green/red *Gobiodon histrio* and the uniformly dark-coloured *Gobiodon* sp. 3 (sensu Herler and Hilgers 2005), are expected to have differential toxicity (Schubert et al. 2003) and resource defence capabilities (Niedermüller et al. 2009). The potential repellent effect of their highly diluted skin mucus on adult *C. austriacus* was additionally tested in a two-channel choice flume experiment.

## Methods

### Study site and sampling techniques

The study was conducted at Dahab, Egypt (28°28′N, 34°30′E), Gulf of Aqaba, Northern Red Sea in April and May 2011. Fishes and corals were collected from one reef flat at a depth of approximately 1.5 m. Adult specimens of *C. austriacus* (total length (TL) > 11 cm) were captured by gill- and hand nets. Breeding pairs of *G. histrio* and *Gobiodon* sp. 3 (TL > 25 mm) were collected from their respective host coral species using low doses of clove oil, diluted with ethanol and seawater. Coral colonies large enough for goby occupation—*Acropora digitifera* (~20-cm diameter; 315 cm^2^ projected planar area) and *A. selago* (~25 cm; 490 cm^2^)—were removed from the substrate with a tungsten steel-wire saw and reattached to the reef using epoxy resin after use to minimise the ecological impact of the experiment.

### Coral predation video experiment

An ex situ experiment was conducted to investigate the influence of gobies on the feeding behaviour of *C. austriacus*. A butterflyfish was given the choice between two equally sized colonies (inhabited by *Gobiodon* vs. empty) of the same species, and bites per visit as well as the number and duration of visits were recorded. Experiments were conducted on both *Gobiodon* species in their respective host coral using two circular tanks (110-cm diameter, 80-cm water depth; 750 l) with a flow-through of ambient sea water. In each of the tanks, a dead coral colony was placed in the centre as a shelter for the butterflyfishes. Butterflyfishes were acclimatised for 12 h and starved for 24 h prior to experiments. To ensure normal behaviour of *Gobiodon* fishes, gobies were also given a minimum of 12 h of acclimatisation within their host colony. The corals were stabilised by inserting them into wide and transparent plastic tubes, which also prevented the butterflyfish from feeding on the stalk of the corals and from hiding underneath them.

To minimise disturbance of butterflyfishes, each tank was sheltered on all sides by white curtains (that still allowed orientation through diffuse sunlight) and equipped with two cameras that enabled tele-close-up recordings in bird’s-eye view of the test corals. Test colonies were always used in pairs (with alternating goby occupation between trials) to avoid differences in colony quality over time. Coral colonies were placed in tanks in the morning after the acclimatisation period of the butterflyfish and recorded for 4 h. Afterwards, corals were removed and the butterflyfishes of the two tanks exchanged. After 15 min of acclimatisation to the new, although identical, tank, the coral pairs were reintroduced into the tanks and recorded for another 4 h. To avoid any site preferences of butterflyfishes from the previous experiment, the axis of orientation of test corals (placed in line with and equidistant from the central shelter coral) was changed by 90° between the two tanks, with the experimentalists’ viewport orientated at right angles to the respective axis. Each butterflyfish was tested twice (in the morning with one coral species and in the afternoon with the other). Each goby pair was always tested in the same colony and the goby occupation state of colony pairs, and their position (to the left versus right of the central shelter coral) was changed between days. Furthermore, goby pairs were exchanged after a few days to avoid starvation while enabling us to test whether the use of gobies from previous trials (with other butterflyfishes) affected the data.

Altogether, 17 trials on 6 colonies (3 pairs) of both *A. digitifera* and *A. selago* with 7 adult breeding pairs of *G. histrio* as well as 9 pairs of *Gobiodon* sp. 3 were analysed. As the motivation of previously starved butterflyfishes might change during the course of a day, the number of morning and afternoon setups was balanced evenly across trials. Some butterflyfishes did not eat at all, took only a few bites during a trial or only explored one of the two coral colonies. As a result, 23 butterflyfishes were used to achieve a total of 17 trials on each coral species.

The duration of a visit was counted as the time that fish spent within one body length of the colony. Visits separated by up to 3 s were counted as a single visit. Similarly, visits of less than 3 s, with the fish merely passing by the colony (without bites), were not recorded. The experiment began once the butterflyfish took at least one bite from each colony and lasted for the remaining time of the total observation time of 4 h. Therefore, the duration of each experiment varied, and data were converted to bites and visits per hour.

### Behavioural observations on coral-defence by *Gobiodon*

Video recordings allowed observation of the reactions of gobies during butterflyfish visits. Although the fixed camera angle did not always capture goby behaviour, video sequences in which one or both of the gobies could be observed were classified. The categories were either (1) “no reaction”, if the gobies remained motionless; (2) “undirected reaction”, when gobies moved between branches or merely shook their body without an obvious movement towards the butterflyfish; (3) “directed reaction”, that is, any kind of movement towards the butterflyfish, with the gobies remaining inside the coral; (4) “dart out reaction”, in which the gobies left the colony and moved towards the butterflyfish; or (5) “physical contact”, in which the gobies darted out of the colony and contacted the butterflyfish. If the goby pair exhibited different categorical behaviours, then the more intensive reaction was noted. The responses of both *Gobiodon* species were summarised as proportions of categories.

### Choice flume experiment

A two-channel choice flume experiment (modified after Gerlach et al. 2007) was conducted to determine whether *Gobiodon* skin mucus has a repellent effect on *C. austriacus*. The test chamber was constructed to be long and wide enough for butterflyfishes to easily swim through and turn around within the channels. The test chamber consisted of a rectangular-shaped upstream section (40-cm long, 24-cm wide, 14-cm water depth) and a trapezoid-shaped downstream section (40-cm long, narrowing to a 4-cm-wide back wall equipped with water run-off hoses). The upstream section was divided into two 12-cm wide flume chambers that each had a central hose entrance for the water inlet and a collimator to homogenise water flow. Periodic dye tests confirmed that the two flumes were clearly separated. A strict laminar flow in the downstream section of the chamber was unattainable because the relatively large butterflyfishes caused water turbulence during trials.

Three 10-l reservoirs, fixed at a height of 1 m above the apparatus, supplied the flume chambers with test water. One reservoir was reserved for pure seawater and two reservoirs alternated between pure seawater and odour-containing water. Water flow was regulated and controlled by tubing clamps with fine flow control set to 1 l min^−1^. This provided an almost constant gravity-driven water flow, as water levels decreased only slightly during testing. Nonetheless, the water flow between the two sides of the flume chamber was similar, and preliminary tests altering flow rate between each side confirmed that butterflyfishes did not prefer a chamber based on flow rate. All tested butterflyfishes spent some time exploring the whole test chamber and then preferred to rest in the downstream section of the test chamber.

For each trial, a single butterflyfish (TL > 11 cm) was introduced into the test chamber, and the whole apparatus was visually isolated using curtains from all sides. A central camera recorded the position of the fish. During acclimatisation, the test chamber was set on circulation with aerated ambient seawater and with the water level of the two seawater reservoirs being aligned by an Archimedes bridge. All fish were given enough acclimatisation and exploration time until they calmly rested in the downstream portion of the chamber for a minimum of 5 min. Thereafter, the circulation was changed to flow-through, the bridge between the reservoirs was removed and, at the 10 l levels, one flume chamber was switched to the 10 l reservoir containing the odour. During preliminary dye tests, the time required for the dye to arrive at the end of the chamber was approximately 2 min (~0.7 cm s^−1^). Therefore, the experiment was started 2 min after the odour was introduced, yielding a total pre-exposure observation time of 7 min, and odour was introduced for an additional 8 min. The experiment was repeated with water sources switched between chambers and the skin mucus of the other *Gobiodon* species once the system and odour reservoir were thoroughly rinsed with fresh seawater, and the focal butterflyfish had been resting in the downstream portion of the test chamber for at least 5 min. Half of the tested butterflyfishes were exposed to mucus from *G. histrio* first, the other half to that of *Gobiodon* sp. 3 first. Altogether, a total of 28 butterflyfishes were tested.

In each trial, 20 l of water ran through the system (10 l per flume chamber). Once the butterflyfish was resting in the downstream part of the test chamber, goby mucus was extracted by placing a single goby into a plastic bag containing 10 ml of seawater. The goby was then gently rubbed between the fingers for 60 s. The induced stress caused the fish to bend its body, squeeze through the fingers and thereby release skin mucus, which was easily recognisable by the water becoming milky and foamy. Thereafter, the fish was released and the 10 ml of odour seawater was mixed with 10 l of seawater, filled into the reservoir and immediately used for the choice flume experiment. For each trial, a new goby and a new plastic bag were used. The maximum amount of released mucus during the 60 s was measured by weighing 10 different adult (TL > 3.8 cm) *G. histrio* specimens to the closest 0.01 g before and after mucus extraction.

### Statistical analyses

All analyses were carried out using IBM SPSS Statistics 20 and 17 as well as Past 2.1 (Hammer et al. 2001).

#### Coral predation video experiment

Differences in butterflyfish foraging behaviour between coral colonies inhabited and uninhabited by the two goby species were investigated using a linear model regression analysis. To identify datasets that could be pooled for the regression analysis, the following tests were conducted to investigate a possible influence of the following factors on the bites and/or visits h^−1^ on inhabited and uninhabited colonies of both coral species: orientation of corals within the tanks (Mann–Whitney U-test); use of corals and gobies from previous trials (ANCOVA on the slope of regressions with trial sequence of the respective coral/gobies as a covariate); differences between goby species pairs (Kruskal–Wallis test); sum of bites and visits between morning and afternoon trials (two-tailed Wilcoxon-sign-test for dependent samples).

Of all these factors, only the sum of bites taken during morning and afternoon trials varied significantly, with more bites taken in the morning trials (*p* < 0.01). Therefore, the linear regression model *y* = *d* + *k*_1_*x_1_ + *k*_2_*x_2_ + *e* for two independent variables (morning/afternoon and status of goby occupation) was used for analysis, where *y* is the value for bites h^−1^, *d* is a constant, *k*_1_ and *k*_2_ are the regression coefficients for the dummy codes *x*_1_ (afternoon trials, which were set to zero) and *x*_2_ (the status uninhabited by a goby, which was set to zero), respectively, and *e* represents the residuals that were tested for normal distribution using Kolmogorov–Smirnov tests. The same statistics were used to investigate differences in visits h^−1^ between inhabited and uninhabited colonies of the two coral/goby species. To determine if coral-associated gobies influence the duration of a single visit and/or the bite rate during these visits, median values were calculated for each trial. Median duration of visits on inhabited and uninhabited colonies was expressed in seconds and the median bite rate per visit as bites s^−1^. For each trial, a Mann–Whitney U-test was carried out for each of these two variables between uninhabited and inhabited colonies. A Wilcoxon-sign-test for dependent samples (two-tailed) was conducted to determine whether there were significant differences amongst the medians of all trials for both coral/goby species.

#### Behavioural observations on coral-defence by Gobiodon

A chi-squared test was used to analyse differences among behavioural categories between both goby species.

#### Choice flume experiment

The seconds that the butterflyfish spent in either one of the two chambers with their whole body were counted using etholog 2.2 (Ottoni 2000) and expressed as proportions of the overall trial time. Wilcoxon-sign-tests for dependent samples (two-tailed) were carried out for the odours from each of the two goby species to determine: (1) avoidance reactions, indicated by differences in proportional residence times before (pre-exposure time of 7 min) and during (8 min) odour release in the downstream chamber and in the flume chamber receiving pure seawater and (2) whether the medians of proportional time spent in the two flume chambers differed during the release of odour. Furthermore, a univariate ANOVA with the fixed factors of goby species and trial order, and with the difference in the proportions of time spent in the two parts of the flume chamber as the dependent variable, was conducted to examine for potential (goby) species-specific differences in the effect of goby mucus and to test whether the preliminary experience from the previous skin mucus of the other goby species caused any significant biases within the second trial. A Levene’s test was used to verify homogeneity of variance, and a Kolmogorov–Smirnov test was conducted to verify normal distribution of the dependent variable.

## Results

### Coral predation video experiment

**Fig. 1:**
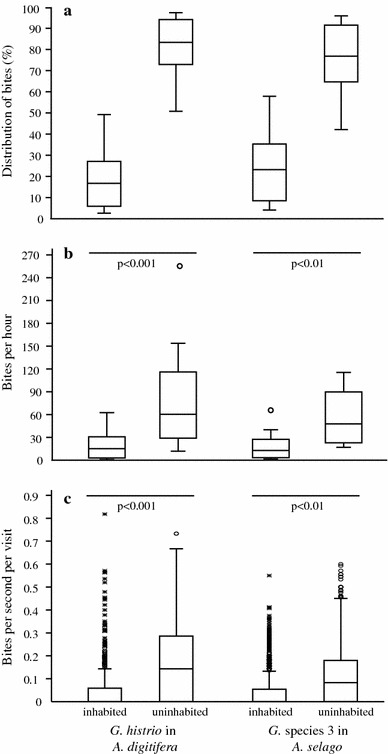
Influence of two coral-dwelling gobies in their respective host colony on the foraging behaviour of the obligate corallivorous butterflyfish *Chaetodon austriacus*. **a** Bites (%) taken by 17 *C. austriacus* on inhabited and uninhabited colonies. **b** Number of bites taken per hour by all *C. austriacus* on inhabited and uninhabited colonies in morning and afternoon trials (difference in day time for *G. histrio* ignored). **c** Bites taken per second and per visit by all *C. austriacus* amongst inhabited and uninhabited colonies. *Box plots* indicate median (*horizontal line*), *upper* and *lower* quartile (*boxes*), and ranges (*whiskers*). *Circles* and *asterisks* indicate outliers. The respective *p*-values are given for data connected by a *horizontal line* above *box plots*

*Chaetodon austriacus* specimens took a total of 8404 bites on all colonies, with 4563 bites observed on *A. digitifera,* and 3841 on *A. selago*. The number of bites taken differed for inhabited and uninhabited colonies for both coral species (Fig. 1a). Standardised values of bites h^−1^ were significantly lower on coral colonies inhabited by both *G. histrio* (*R* ^2^ = 0.387, *F*_2,33_ = 11.425, *p* < 0.001) and *Gobiodon* sp. 3 (*R* ^2^ = 0.323, *F*_2,33_ = 8.858, *p* < 0.01) (Fig. 1b), with the residuals for both regressions distributed normally. The reduction in bite rate (calculated from the regression analysis) due to goby occupation averaged 80 % for coral colonies inhabited by *G. histrio* (morning: 62 %, afternoon: 98 %) and 66 % for coral colonies inhabited by *Gobiodon* sp. 3 (morning: 64 %, afternoon: 68 %). In contrast, the number of visits per hour of *C. austriacus* between inhabited and uninhabited colonies of both goby species was not significantly different.

Whereas the median bite rate of butterflyfishes per visit (Fig. 1c) was significantly higher on uninhabited colonies (*G. histrio*: *p* < 0.001; *Gobiodon* sp. 3: *p* < 0.01), the median duration of visits was similar. In 16 out of 17 *G. histrio* trials, the median bite rate per visit was significantly higher on the uninhabited colony. Interestingly, the highest bite rate (0.82 bites s^−1^ visit^−1^) was observed on an inhabited colony (Fig. 1c). In *Gobiodon* sp. 3 trials, only 10 out of 17 butterflyfishes exhibited a significantly higher bite rate on the uninhabited colony; moreover, none showed a significantly higher bite rate on the inhabited colony. In contrast, visit duration revealed individual differences among butterflyfishes, with some individuals even exhibiting significantly longer visits on inhabited colonies. Within *G. histrio* trials, 47 % of butterflyfishes showed significant differences in visit duration, with 63 % out of these spending significantly more time per visit at the uninhabited colonies. In trials with *Gobiodon* sp. 3, 35 % of all butterflyfishes exhibited significant differences in visit duration. However, the proportion spending more time at the uninhabited colonies was similar (67 %).

### Behavioural observations on coral-defence by *Gobiodon*

**Fig. 2:**
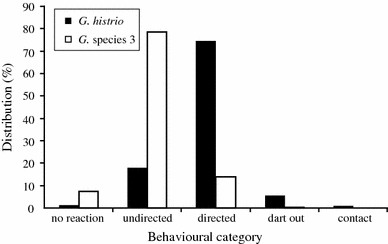
Distribution of behavioural responses (%) displayed by *Gobiodon histrio* (*black bars*, *n* = 460) and *Gobiodon* sp. 3 (*white bars*, *n* = 508) in response to visits by the corallivorous butterflyfish *Chaetodon austriacus*

A total of 660 butterflyfish visits to colonies inhabited by *G. histrio* and 975 to those inhabited by *Gobiodon* sp. 3 were analysed. Due to the more complex structure of *A. selago* colonies, the number of visits in which interactions could not be recorded was higher for *Gobiodon* sp. 3 than for *G. histrio* (467 vs. 200 visits without behavioural observations). Since inhabited *A. selago* colonies were visited more frequently, the total number of visits with behavioural observations were similar for *G. histrio* (*n* = 460) and *Gobiodon* sp. 3 (*n* = 508). *G. histrio* exhibited stronger agonistic behaviour than *Gobiodon* sp. 3 (Fig. 2). In most cases (79 %), *Gobiodon* sp. 3 reacted to butterflyfishes by undirected movements. These were mostly movements and body-bending turns between branches as well as shaking movements of body and fins. In 14 % of cases, *Gobiodon* sp. 3 reacted with a directed movement towards butterflyfishes, but on only one occasion did the goby dart out of the coral. In contrast, *G. histrio* exhibited undirected behaviour in 18 % of cases and typically reacted by directed movements (75 %) towards butterflyfishes. The latter category involved following the butterflyfish, guarding the approached area and also darting towards the foraging area of the butterflyfish in the coral colony. In 5 % of the cases, the goby darted out of the colony towards the butterflyfish. In 1 % of the observations, physical contact between the goby and butterflyfish occurred, a behaviour never observed with *Gobiodon* sp. 3. Finally, the “no reaction” category was less frequent in *G. histrio* (1 %) than in *Gobiodon* sp. 3 (7 %). These behavioural categories differed significantly between the two gobies (χ^2^ = 80.2, *p* < 0.001; categories “no reaction”, “dart out” and “contact” pooled as one).

### Choice flume experiment

This experiment tested whether *Gobiodon* skin mucus has a repellent effect on corallivorous butterflyfishes. In general, goby mucus caused butterflyfish to abandon the downstream portion of the flume chamber. Butterflyfish spent significantly less time in the downstream part of the chamber after the odour reached this section (median residence time 100 % vs. 68 %, *df* = 27, *p* < 0.001), resulting in a significant increase in occupation of the portion of the flume chamber without goby odour (0 vs. 25 %, *df* = 27, *p* < 0.001). The maximum amount of mucus released by a single goby was 0.08 g wet weight (approximated to a volume of ~0.1 ml) during 60 s of gentle rubbing. The 10 ml of seawater containing *Gobiodon* mucus (~0.1 ml) was diluted with 10 l of seawater in the reservoir. This seawater was further diluted as it intermixed with seawater from the second flume chamber in the downstream section of the test chamber. Based on the initial amount of mucus and the flume volume, butterflyfish reactions to goby skin odour in the downstream part of the chamber were thus caused by a dilution of at least 1:200,000.

**Fig. 3:**
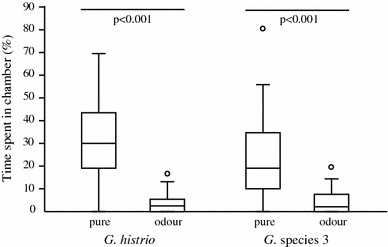
Median time (%) spent by all 28 *Chaetodon austriacus* in flume chambers with either ambient seawater (pure) or ambient seawater containing *Gobiodon* odour (odour) of the two coral-dwelling gobies (*G. histrio* and *Gobiodon* sp. 3). *Box plots* indicate median (*horizontal line*), *upper* and *lower* quartile (*boxes*), and ranges (*whiskers*). *Circles* indicate outliers. The respective *p*-values are given for data connected by a *horizontal line* above *box plots*

During the 8 min of odour release, butterflyfishes spent significantly less time (*df* = 27, *p* < 0.001) in the flume chamber containing the odour of both *G. histrio* and *Gobiodon* sp. 3 (Fig. 3). The previous experience of butterflyfishes from the first trial had no significant effect on their choice during the second trial. Furthermore, the effect of the mucus of the two goby species differed significantly (*F*_3,52_ = 4.501, *p* < 0.05). Although the time that butterflyfishes spent in the flume chamber containing the *Gobiodon* odour was similar for both goby species, those fishes exposed to *G. histrio* odour spent less time in the downstream part of the chamber (mixed water) and more time in the flume chamber with pure seawater than butterflyfishes exposed to odour from *Gobiodon* sp. 3. Furthermore, in trials using *Gobiodon* sp. 3 odour, five butterflyfish remained in the downstream part of the chamber for the entire experiment, whereas only one of 28 butterflyfish remained in this location when exposed to *G. histrio* odour. In summary, the highly diluted odours of both species caused significant avoidance reactions in *C. austriacus*.

## Discussion

Coral-dwelling organisms contribute to the fitness of their host coral colony by various mechanisms, including resource defence against coral predators (Weber and Woodhead 1970; Godwin and Fautin 1992; Pratchett 2001; Porat and Chadwick-Furman 2005; Stewart et al. 2006). Although it is known that coral-dwelling gobies of the genus *Gobiodon* are highly dependent on the shelter of healthy *Acropora* corals, it was unclear whether the host corals also benefit from these associations. Our hypothesis that *Gobiodon* exhibits resource defence against corallivorous butterflyfishes was clearly confirmed by these experiments: the bite rate of *C. austriacus* was significantly lower on inhabited colonies. The choice flume experiment suggests a repellent effect of *Gobiodon* skin mucus on butterflyfish, which showed significant avoidance reactions.

Despite a clear interspecific difference in behavioural interactions with butterflyfishes and in the effect of odours, both *Gobiodon* species reduced coral predation rates. One species, *G. histrio*, is a superior competitor within its genus (Munday et al. 2001). This suggests that it readily exhibits agonistic behaviour against con- and heterospecific fishes. Such behaviour would also be facilitated by the wider branching pattern of the typical host corals of *G. histrio* (e.g., *A. digitifera* or *A. gemmifera* in the Northern Red Sea), whereas the larger-growing (up to 1-m diameter in the Northern Red Sea) and more finely branched *A. selago* would be more difficult to defend. In line with this assumption, *G. histrio* exhibited significantly more directed behaviour towards butterflyfishes in this study, whereas *Gobiodon* sp. 3 responded mostly by undirected body movements. The highly directed behaviour of *G. histrio*, combined with stronger avoidance reactions in butterflyfishes towards its odour, resulted in a greater reduction of coral predation rates compared to *Gobiodon* sp. 3. This implies species-specific differences in resource defence capabilities, suggesting different effects on the foraging activity of butterflyfishes among inhabited *Acropora* species. Nonetheless, both gobies significantly reduced coral predation, even though *Gobiodon* sp. 3 rarely displayed agonistic behaviour. Accordingly, odour apparently at least partially supports resource defence by acting as a predator deterrent.

The deterrent effect of goby odour in the flume experiment was uncoupled from the fish and its behaviour as well as from butterflyfish feeding. It is, however, very likely that avoidance reactions imply that a butterflyfish would also refuse to feed in this area. Lassig (1981) induced such food refusal reactions, suggesting a potential repellent effect of *Gobiodon* skin mucus on butterflyfishes. The dosage in his experiment, however, was very high (entire epidermis scrapings of one goby in 50 ml of seawater) and killed butterflyfishes within minutes. Those reactions are therefore not particularly applicable to natural field conditions. Determining and simulating the exact concentration and dosage of minute discharges of goby skin mucus into the water column upon interactions with butterflyfishes are difficult. Importantly, the much greater dilution (min. 1:200,000) in the present study also resulted in avoidance reactions by butterflyfish. When manipulated to extract skin mucus for avoidance experiments, gobies bent their entire body. Similar movements were observed during goby–butterflyfish interactions. Gobies darted towards and then turned away from the visitor, or they swam and circled between branches while shaking their bodies and exhibiting fin movements. Both these patterns included body-bending movements. Agonistic behaviour alone, however, cannot explain the reduced predation rate on colonies inhabited by *Gobiodon* sp. 3, suggesting that a combination of goby behaviour and chemical defence is responsible for butterflyfish avoidance.

In the feeding preference experiment, butterflyfish bite rates were sometimes high on inhabited colonies (Fig. 1c), which were observed when *Gobiodon* was not present at the exact area in the coral colony upon which the butterflyfish was feeding. This suggests that the effect of gobies is spatially constrained within coral colonies, and it is of course unclear how strong the chemical deterrent would be in high current situations. The effect measured in the ex situ experiment (minimal water flow and medium-sized (~400 cm^2^) coral colonies) might therefore decrease with increasing colony size and current in the natural environment. On the reef, the likelihood of *Gobiodon* encountering a foraging butterflyfish on their host coral should similarly decline with increasing colony size. Niedermüller et al. (2009) investigated the influence of *Gobiodon* occupation on the foraging activity of *C. austriacus* in the field. Those authors found a trend among butterflyfishes to prefer uninhabited corals; goby occupation rates were lower among selected than among available colonies in all size classes (0− > 800 cm^2^). *Chaetodon austriacus* prefers large-sized *A. selago* colonies, although these were usually inhabited by *Gobiodon* sp. 3. This pattern reflected the preference of both butterflyfishes and gobies for large colonies (>600 cm^2^) for food and habitat, respectively. Note that *A. selago* might be generally more attractive to coral feeders because the polyp tentacles are frequently extended during the day (personal observations). In contrast, the generally smaller *A. digitifera* and other coral species were significantly avoided if inhabited by *G. histrio*. The present study demonstrates that *Gobiodon* sp. 3 also exhibits resource defence that results in reduced predation rates, at least for medium-sized *A. selago* colonies. The potential of resource defence might therefore be limited by coral inter-branch spacing and size. Such colony shape- and size-based dependence might explain the influence of colony size on selective corallivory among (Gore 1984) and within (Cole and Pratchett 2011b) acroporid species.

The bite rates in the experimental setup varied considerably between individual *C. austriacus* and were generally lower (13–270 bites h^−1^) than the average bite rate observed in the field (362 bites h^−1^; Alwany et al. 2003). In general, obligate corallivorous butterflyfishes take 400–700 bites h^−1^ throughout the day (Tricas 1989; Gregson et al. 2008), with an average of 420 bites h^−1^ (Rotjan and Lewis 2008). The low experimental bite rates probably reflect the artificial conditions in combination with providing only two coral colonies for food. Chaetodontidae naturally avoid such chronic predation by foraging across multiple colonies within their territory (e.g., Roberts and Ormond 1992). Furthermore, bite rates might have been reduced by short-term polyp retraction as a result of butterflyfish predation (Gochfeld 2004). Our observation that *C. austriacus* did not visit uninhabited colonies more often or longer might therefore be an artefact of the low number of coral colonies offered as food. In the field, where coral availability is much greater, butterflyfishes would most likely choose other colonies upon being confronted with resource defence by *Gobiodon* spp. and indeed make more visits to and spend a longer time foraging on uninhabited colonies (Lassig 1981). Similarly, under field conditions, there is no evidence for the diurnal feeding variation of butterflyfishes observed in this study. This implies that the hunger resulting from forced starvation increased feeding rates in the morning during the experiment.

Despite the low bite rates measured here, *G. histrio* decreased the average bite rate by 80 % and *Gobiodon* sp. 3 by 66 %. Accordingly, uninhabited coral colonies suffered from higher predation rates than inhabited colonies. The diet of *Gobiodon* compromises a broad range of items, mostly zooplankton and filamentous algae, but facultative corallivory has been documented for some species (Riedlecker and Herler 2009; Brooker et al. 2010). Nonetheless, this facultative corallivory is most likely outweighed by the impact of obligate coral feeders, whose tissue and polyp removal implies significant fitness consequences for corals (e.g., Cole et al. 2011, 2012). By reducing the predation rate through resource defence, the association between coral-dwelling *Gobiodon* and their host coral colonies might therefore be regarded as mutualistic. On the other hand, the allocation of energy and time to resource defence might only compensate for potential negative effects from permanent coral occupation (i.e., facultative corallivory, reduction of food/oxygen amount, toxic effects of skin mucus). Although energy trade-offs between regeneration, coral growth and fecundity have been documented for many corals (e.g., Meesters et al. 1994; Vanveghel and Bak 1994; Hall 2001), the influence of chronic and repeated tissue grazing on acroporid growth rates remains largely unknown. Further experiments are necessary to assess the impact of chronic butterflyfish corallivory on acroporid fitness and growth rates. In the presence of butterflyfish predation, goby-inhabited colonies have an advantage. Nonetheless, whether and to what extent facultatively corallivorous and coral-associated organisms such as species of *Gobiodon* influence the fitness of host colonies, in the absence of corallivory, remains unknown.

This study clarified the influence of two *Gobiodon* species on the feeding preferences of butterflyfishes by controlling for any confounding variables of in situ observations. Future research is required to determine the ecological consequences and its significance in the field, especially with respect to the relative choices of butterflyfishes between generally preferred but occupied versus less preferred but unoccupied coral species. Investigations of butterflyfish feeding should therefore take the *Gobiodon* occupation status of *Acropora* colonies into account. As occupation rates of common host corals never reach 100 % (4.1–71.2 %, average: 47.1 %) in the investigation area (Schiemer et al. 2009), attention should be given to investigate disproportional feeding activities of corallivores among same-sized colonies of host coral species. Although some information is available about the composition of skin mucus (Hashimoto et al. 1974), the specific compound that causes the observed deterrent effect in butterflyfishes has yet to be identified. Knowledge about its concentration and effective dosage upon discharges would allow for conclusions about its repellent effect further away from the host colony or under different water flow conditions. We also lack information about trade-offs in relation to the costs of producing this crinotoxic mucus in *Gobiodon*.

In summary, this study demonstrates that coral-dwelling gobies of the genus *Gobiodon* influence the feeding behaviour of an obligate corallivorous butterflyfish. These gobies reduce the predation rate on their host colony through agonistic behaviour and the repellent effect of their skin mucus; such mucus induces strong avoidance and most likely food refusal reactions among butterflyfishes. Corallivorous butterflyfish fed more on uninhabited colonies, which thus experienced increased predation rates. The effectiveness of resource defence by coral gobies is most likely constrained by coral colony inter-branch spacing and colony size. Since the average colony size is likely to decrease due to increased bleaching frequency (Hoegh-Guldberg 1999; McClanahan et al. 2008), the impact of coral-dwelling gobies on butterflyfish feeding may increase. This will further intensify predation on uninhabited coral colonies or even lead to a shift of butterflyfish feeding preferences to coral species/taxa without goby defence. Although butterflyfishes might not threaten the survival of colonies, goby-inhabited colonies are expected to have accelerated growth rates, more polyps, higher reproductive capacities and an increased resistance to other stressors. Such fitness advantages for host colonies would directly benefit gobies by increasing space available for feeding and breeding.
